# “Better to Die Than Take These Medicines”: A Community-Based Qualitative Study on the Determinants of Treatment Loss-to-Follow-Up in Tuberculosis Patients in District Faridabad, Haryana, India

**DOI:** 10.7759/cureus.25030

**Published:** 2022-05-15

**Authors:** Anwita Khaitan, Sanjay K Rai, Anand Krishnan, Sanjeev K Gupta, Shashi Kant, Gopi C Khilnani

**Affiliations:** 1 Centre for Community Medicine, All India Institute of Medical Sciences, New Delhi, New Delhi, IND; 2 Pulmonary Medicine and Sleep Disorders, All India Institute of Medical Sciences, New Delhi, New Delhi, IND

**Keywords:** community-based, determinants, qualitative research, side effects, alcohol use, default, loss-to-follow-up, tuberculosis

## Abstract

Introduction

India is the biggest contributor to the global incidence of tuberculosis (TB). A major reason behind the persistently high incidence of TB in India is treatment loss-to-follow-up (LTFU). The consequences of LTFU include continuous transmission to uninfected individuals, drug resistance, and a higher rate of death in incompletely treated patients. It is a significant hurdle to making India ‘TB-Free’ by 2025. Hence, we conducted a community-based qualitative study to understand the determinants of treatment of LTFU in TB patients in the Faridabad district of Haryana, India.

Methodology

We enrolled TB patients who had completed treatment as well as those who had been LTFU. We also enrolled National Tuberculosis Elimination Programme (NTEP) functionaries, healthcare providers, family members, and community members. In-depth interviews (IDIs) and focus group discussions (FGDs) were conducted to understand stakeholders’ perceptions of reasons for LTFU. The grounded theory approach was used with inductive analysis. Data were triangulated from stakeholders’ interviews. Themes and sub-themes were identified. A Health Belief Model for TB treatment completion was developed.

Results

Fifty-eight IDIs and four FGDs were conducted between May-June 2018. The major themes influencing the treatment of LTFU which emerged from the analyses were - the role of external motivators, regular use of alcohol, lack of/or inappropriate knowledge related to treatment, lack of family support, and side effects of anti-tubercular drugs, and a poor experience with the health system. Stigma was not found to be a major determinant - in the few cases that it affected treatment, it spurred treatment completion rather than LTFU.

“*I completed the course with great difficulty. Then they started it again! […] I said-Sorry, sir, I can’t go through this again. It’s better to die once than to die a thousand deaths.”* - Fifty-one-year-old male patient who was lost-to-follow-up on re-treatment.

Discussion

This study was a comprehensive multi-stakeholder qualitative undertaking to identify the determinants of LTFU. Our qualitative approach explained the associations between LTFU and certain factors (e.g.: alcohol use, side effects, etc.) found in previous quantitative studies. The strength of this study was that we ensured participation by patients as well as all district-level stakeholders from the national health programme, which no previous qualitative study on the treatment LTFU in India had achieved. The entire qualitative analysis was done manually and in Hindi (the language in which interviews were conducted). Hence, no data were lost in translation. The limitation was that its findings were specific to the study area and study population, as is the case with all qualitative studies.

Conclusion

All healthcare providers should be sensitised to the determinants of treatment LTFU, so that they can pay special attention to at-risk patients and take appropriate steps to prevent LTFU. For instance, patients with a pattern of regular alcohol use should be counselled and may be referred to deaddiction centres, with the continuum of care maintained. The journey from tuberculosis diagnosis to treatment completion is often extremely traumatic for the patient. The onus to successfully complete treatment lies not with the patient alone, but with the health system as well.

## Introduction

India is the biggest contributor to the global burden of tuberculosis (TB), accounting for 27% of the 10 million incident cases, 24% of the 558,000 incident multi-drug resistant (MDR) cases, and 27% of the 1.5 million TB-related deaths annually [1]. The National Strategic Plan (NSP) for TB Elimination 2017-2025 aims to bring down the TB incidence rate to less than 44 per 100,000; the prevalence rate to less than 65 per 100,000; and the mortality to less than three per 100,000 by 2025 [2].

In 2018, at the End TB Summit held in New Delhi, the Government of India declared the vision of having a 'TB-Free' India by achieving the UN Sustainable Development Goals’ TB-related targets five years in advance of the target year of 2030, hence “eliminating tuberculosis from India” by 2025 - the most ambitious target yet [3]. To achieve this target, no patient who comes under the care of the National Tuberculosis Elimination Programme (NTEP) should fall through the cracks. In reality, however, one-third of the patients being treated in the public health sector are lost between care-seeking and successful cure at multiple levels [2]. One such level for a diagnosed patient is treatment loss-to-follow-up (LTFU), defined as discontinuation of treatment for one or more month(s) after receiving it for at least one month. It was previously termed as ‘treatment default’ [4].

In spite of the provision of free-of-cost medicines under the Directly Observed Treatment-Short course (DOTS), the reported LTFU rates are high, ranging between 15-25% [5-10]. Quantitative studies have reported many factors that are associated with LTFU. However, there is a paucity of qualitative studies in India that provide an understanding of the causal pathways of a suspected determinant leading to LTFU. The few existing qualitative studies had restricted themselves to reporting the viewpoints of either the patients or their family members or the DOTS providers [9,11-15]. Other studies, conducted outside India may have low transferability to our setting [16-19]. Therefore, the primary objective of this qualitative study was to identify the determinants of treatment LTFU in tuberculosis patients residing in district Faridabad, Haryana through the inclusion of a wide range of stakeholders, and to explain the causal pathways through the Health Belief Model (HBM) - a framework widely used to explain health-related behaviours such as treatment adherence. [20].

## Materials and methods

Study setting and study participants

We carried out our study in the Ballabgarh block of district Faridabad in the North Indian state of Haryana. In our study setting, the treatment LTFU rate has been reported to be 11-14% [6,15]. The district has nine Tuberculosis Units (TUs) - a TU is the nodal point of the NTEP at the sub-district level, catering to a population of 200,000 (with a range of 150,000-250,000). Out of the nine TUs, two were purposively selected - TU Ballabgarh and TU Mohna. TU Ballabgarh, located within the premises of sub-district hospital Ballabgarh, caters to a primarily urban population. TU Mohna, located at the Primary Health Centre (PHC) Mohna, serves an almost exclusively rural population. A total of five Peripheral Health Institutions (PHIs) and three Designated Microscopy Centres (DMCs) cater to the population of these two TUs.

We included participants with a diverse profile, who were involved in TB care either directly or indirectly. These included:

Tuberculosis Patients

Patients who were LTFU while on treatment, as well as those that had completed the treatment (Outcome - Treatment complete/ Cure), were included. Those patients were eligible for inclusion who were aged 18 years or older, whose treatment had been initiated at TU Ballabgarh or TU Mohna between 1st October 2016 and 30th September 2017, and who belonged to one of the following two groups - 

1. LTFU patients: Patients whose outcome had been declared as LTFU, or those initiated on ‘treatment after LTFU' with or without treatment outcome being declared. 2. Completed treatment: Patients not on 'treatment after LTFU' and whose outcome had been declared as ‘Treatment Complete’ or ‘Cure’.

A line list of eligible patients was prepared from available TB registers of the two TUs. All eligible LTFU patients who were living and had not emigrated elsewhere were approached for participation in the study - hence no sampling was done in the case of LTFU patients. Ten eligible patients who had completed treatment were purposively selected from the line list and were approached for participation - five from TU Ballabgarh, and five from TU Mohna. The numbers obtained were adequate for data saturation to be achieved.

Healthcare Providers

District TB Officer, Medical Officers (TB In-charge), Senior Treatment Supervisors, Senior TB Laboratory Supervisor, Lab Technologists, TB Health Visitors, Health Supervisors, Multi-Purpose Health Workers (Male and Female), DOTS providers were purposively sampled from the two TUs of district Faridabad.

Community Members

Panchayati Raj Institution (PRI) members and community members (male and female) were purposively sampled from district Faridabad.

Development of tools

*In-Depth Interview (IDI) Schedule* The IDI schedule had two sections. The first section was to collect socio-demographic details. The second section comprised 35-39 interview questions (depending on the stakeholder for whom it was customised). These questions were open-ended and were based on the HBM and health-seeking behaviour. Each question had some optional probes to facilitate the interview process, but no prompts (to avoid introducing bias in the findings). None of the questions alluded to any themes previously identified in literature, except one question. This question was on the perceived impact of alcohol use on treatment adherence - based on literature review and experience in the study setting, the authors felt it merited detailed discussion with the stakeholders. These interview schedules were pre-tested on patients and on health care providers (who were not included in the study participants) and modified accordingly. *Focus Group Discussion (FGD) Guide* The topic guide for the FGDs was developed with a focus on socio-cultural beliefs related to TB, possible causes of LTFU, and possible solutions. It comprised three sections - The first was to collect socio-demographic details of the participants. The second section comprised FGD topics. The third section was to construct the sociogram. 

Data collection

Interviews and FGDs were conducted in Hindi by the first author (AK) - a Hindi-speaking Community Medicine MD resident. She was trained and supervised in the data collection and analysis process by the co-authors (KA, SKR), who are senior faculty members in a tertiary care referral teaching hospital with extensive experience in qualitative research.

After taking a telephonic appointment from the potential patient participant, IDIs were conducted face-to-face at their homes. Each interview was started after explaining the purpose of the study, providing a bilingual participant information sheet (PIS), and obtaining written informed consent from the participant. Since many sensitive topics would be discussed, an effort was made to ensure privacy as far as possible within the household. Alternatively, a culturally appropriate nearby location for the interview would be chosen, where privacy could be ensured. Similarly, after taking a telephonic appointment with the potential key informant, KIIs were conducted face-to-face in their offices, after explaining the study's purpose, providing the PIS, and obtaining written informed consent.

FGDs were conducted in the primary health centres (PHCs) where DMCs were located. They were conducted in Hindi with a single moderator (AK), two note-takers, and one person who made the sociograms. The study's purpose was explained to the group, and individual-level written informed consent was obtained. An audio recording was done using a digital recorder. Field notes were made during and especially after each interview and FGD. 

Data analysis

The data analysis was based on the grounded theory of qualitative research and was inductive in its approach. Devanagari transcription of audio recordings was done. Transcripts were analysed by three analysts (AK, KA, SKR) using the open-coding technique. All analysts were fluent in both English and Hindi. Hence no data were lost in translation. During the preliminary reading of the transcripts, relevant portions of the text were free-listed. Textual annotations were made next to the free-listed items. Textual annotations were summarised on a cover page and used to develop the list of recurring themes and sub-themes. Inductive codes were developed for these recurring themes, and all transcripts were catalogued according to this preliminary coding system. The codes were compiled into sub-domains and then overall domains. The coding system was modified and finalised based on an iterative analysis of the transcripts. Throughout this process, the analysts kept identifying similarities and differences in the perceptions across various stakeholders. Then, by triangulating data across different stakeholders and data collection techniques, the final list of domains and sub-domains was prepared. Profiles of potential LTFU and potential treatment completers were developed. The HBM for treatment completion was constructed. All identified themes were arranged into various components of HBM.

Ethical approval

The study was approved by the Institute Ethics Committee of the All India Institute of Medical Sciences, New Delhi (Ref. No. IECPG-460/29.11.2017). Written informed consent was obtained from all the participants.

## Results

Stakeholders’ characteristics

The prepared line list had 50 individuals that were LTFU or were on treatment after LTFU and met the eligibility criteria. Out of the 27 who were alive, 20 were traceable and enrolled in the study. Sixteen of these participants were men. Their mean age was 40 years, with a range of 18 to 70 years (Table 1). The mean duration of each IDI was 68 minutes.

**Table 1:** Sociodemographic characteristics of TB patient-participants from two Tuberculosis Units (TUs) of district Faridabad, Haryana, India. SD - Standard Deviation; IQR - Inter-quartile range; INR - Indian National Rupee (76.3 INR = 1 USD w.e.f. 04 May 2022).

	Treatment LTFU (N=20) n (%)	Successful treatment (Treatment completion/Cure) (N=10) n (%)
Mean age in completed years (SD)	39 (14)	38 (18)
Median age in completed years (IQR)	42 (23-50)	31 (22-57)
Gender		
Male (%)	16 (80)	5 (50)
Female (%)	4 (20)	5 (50)
Marital status		
Never married	6 (30)	1 (10)
Married	10 (50)	7 (70)
Separated or divorced	2 (10)	0 (0)
Widowed	2 (10)	2 (20)
Level of completed education		
Illiterate	6 (30)	2 (20)
Primary	9 (45)	2 (20)
Secondary	3 (15)	2 (20)
Higher secondary	1 (5)	2 (20)
Graduate	1 (5)	2 (20)
Type of Profession		
Unskilled	6 (30)	2 (20)
Semi-skilled	14 (70)	7 (70)
Skilled	0 (0)	1 (10)
Median monthly per capita income per capita in INR (IQR)	9000 (6000-15000)	8500 (5750-14250)
Source of treatment		
Public	17 (85)	8 (80)
Private	3 (15)	2 (20)
Tuberculosis Unit (TU)		
TU Mohna (Rural)	13 (65)	5 (50)
TU Ballabgarh (Urban)	7 (35)	5 (50)

There were 652 eligible patients (444 from TU Ballabgarh and 208 from TU Mohna) who had successfully completed the treatment. We purposively selected ten of these 652 treatment completers (five from TU Ballabgarh and five from TU Mohna). Half of them were men. Their mean age was 38 years, with a similar range of 18 to 70 years (Table 1). The mean duration of each IDI was 41 minutes. 

A total of 28 key informant interviews (KIIs) were conducted. The mean duration of each KII was 47 minutes.

Two FGDs were conducted with community members: first with 11 women, and second with eight men. Two FGDs with 10 DOTS providers each were also conducted. The mean duration of each FGD was 45 minutes.

Thus, a total of 58 IDIs and four FGDs were conducted (Table 2). One to three attempts were required to contact each interviewee and conduct the IDI/KII.

The transcription of these IDIs' and FGDs' audio recordings took approximately 660 person-hours and proofreading of the transcripts took around 110 person-hours. Data collection and analysis took approximately six months.

**Table 2:** Stakeholders with whom in-depth interviews (IDIs) and focus group discussions (FGDs) were conducted in two Tuberculosis Units (TUs) in District Faridabad, Haryana, India.

In-Depth Interviews (IDIs) and Focus Group Discussions (FGDs)	Number
IDIs with TB patients who were lost-to-follow-up (LTFU) or were on treatment after LTFU	20
IDIs with TB patients with successful outcome (Cure/Treatment Complete)	10
IDI with District Tuberculosis Officer	1
IDIs with Medical Officer – TB Control	2
IDIs with Health Supervisors at Primary Health Centres (PHCs) with Designated Microscopy Centres (DMCs)	3
IDIs with DMC Laboratory Technologists	3
IDIs with Peripheral Health Institution (PHI) Health Visitors	2
IDIs with DOTS Providers	2
IDIs with PHC Medical Officers-In-Charge	5
IDIs with Panchayati Raj Institute (PRI) Members	3
IDI with Senior Tuberculosis Laboratory Supervisor	1
IDIs with Senior Treatment Supervisors	2
IDIs with Multi-Purpose Health Workers (Male)	4
FGD with female community members	1
FGD with male community members	1
FGDs with DOTS providers	2

Determinants of tuberculosis treatment loss-to-follow-up - major domains

The domains/themes that emerged after the final analysis of IDIs and FGDs were as follows:

External Motivators

Patients who had successfully completed the treatment dwelled less on the side effects of medicines. They focused more on avoiding the perceived threats of discontinuing the treatment prematurely, and reaping the benefits of treatment completion. The perceived threats included: death, spreading the disease to their loved ones, and longer duration of repeat treatment course. The perceived benefits included: prospect of cure, end of their isolation period, and improved prospect of marriage, if unmarried. For few patients the perceived benefit also included resumption of tobacco and alcohol use. Most stakeholders felt that patient’s motivation level was one of the strongest determinants of treatment completion.

“For family, I needed to get cured, to stay with my children. (I) would never want them to get the disease. The main purpose was that no one else in the household should get it, and I should also not get it again.” - IDI-22 (Twenty-nine-year-old female with successful outcome)

Regular Use of Alcohol

Most stakeholders felt that alcohol was an important determinant of LTFU. Some key informants went so far as to state that a patient who had a history of regular alcohol use be considered a “*lost cause*”, who would inevitably abandon the treatment regimen. Some patients were convinced that medicines and alcohol taken together would result in an adverse “*reaction*”, and it was easier to choose alcohol over the medicines. Some patients who could not abstain from alcohol decided to abandon the treatment and resumed alcohol use. Some patients had a fatalistic attitude towards life, and opined that “*they would rather die drinking than taking the medicines*”. LTFU was often preceded by many episodes of missed dose due to their state of inebriation.

“They think that if they take medicines, they have to stop drinking. Thus, those who are addicted, they stop taking medicines because of alcohol. Then when their condition worsens, they come begging for treatment, promising they won’t drink anymore. We resume the treatment, and they’ll abstain from drinking for a month. Then they’ll go to some wedding function or the other, and start drinking again. And again, they stop taking medicine.” - FGD-4 (DOTS provider)

Knowledge

Majority of the stakeholders felt that formal education level of the patient was less important compared to counselling for treatment completion. Some patients who had felt better after a few months of treatment thought that they were cured. Others, who didn’t get symptomatic relief, or felt worse off because of the side effects of medicines, thought that the treatment had failed. In either case, they left the treatment prematurely in spite of knowing the prescribed duration of the treatment. Another noteworthy finding was that a few participants who had were LTFU reported that they had left treatment because they thought that negative sputum smear results (at the end of intensive phase) meant that they were cured.

“The reason behind default is that the patient takes the medicine for two months and then thinks that he is completely fine. […] But an educated family knows that a six-month course needs to be completed, only then will they be cured. And the disease won’t happen again.” - FGD-4 (DOTS provider)

Family Support

Family support emerged as one of the key determinants of treatment completion. Most patients felt that family support was needed for proper and regular meals and reminders to take medicines on time. Family encouragement also helped them to continue taking medicines in spite of their side effects. Members of the extended family sometimes helped financially, both directly or indirectly. Physically weak middle-aged and elderly patients got family support for activities of daily living such as bathing and getting dressed. All stakeholders opined that the most significant contribution of family members was to provide emotional and moral support.

“My husband used to keep telling me that regardless of if we are fighting, no matter what happens, even if we have to go in the rain to get the medicines, I must not stop taking the medicines. He used to keep telling me that if anything were to happen to me, then he would be left with no one.” - IDI-21 (Thirty-four-year-old female with successful outcome)

Side Effects of Anti-Tubercular Drugs

Side effect of the anti-tubercular (ATT) drugs was identified as a barrier to treatment completion by the majority of the healthcare providers. Some patients even felt that it was *“better to die than take these medicines”*. Almost half of the LTFU patients stated that the absence of any provision of medicine to alleviate the side effects had led them to leave the treatment.

“When the doctor used to give medicines, after I took the red capsule, I used to vomit. The vomit was red, like the medicines. […] I completed the course with great difficulty. Then it started again. I buckled at the prospect. […] I said, “Sorry, sir, I can’t go through this again. “Better to die once than to die many deaths.” - IDI-11 (Fifty-one-year-old male treatment LTFU)

Interaction with Health System

The interface between patients and the health system was the healthcare provider. Almost all stakeholders felt that the behaviour of healthcare providers, particularly the DOTS provider, was a crucial determinant of the treatment completion. Some LTFU reported that their DOTS provider had spoken with them ‘very rudely’. Though other factors e.g. regular alcohol use, were also present which made them vulnerable to LTFU, rude behaviour of healthcare provider was identified as a trigger for abandoning the treatment.

The majority of key informants and treatment completers said that frequent counselling, prompt and early action if a patient misses one or two doses helps avert treatment LTFU.

Most patients felt that the health system should provide free medicines to relieve the side effects. Dietary support from the health system was also a recurring point of discussion in the interviews with key informants. Healthcare providers interviewed in our study felt that free rations would help patients adhere to treatment, since it would improve their overall health status and increase their tolerance towards side effects. Healthcare providers opined that the money provided for dietary support could be misused, especially by those who were addicted to substances such as alcohol.

Some key informants and patients felt that the side effects of the daily regimen compared to the intermittent regimen were lesser, possibly due to the lower pill burden per dose. This presumably enabled them to achieve treatment completion.

“[The health-worker] spoke to me in such a manner that I never went back to him. He said, “Am I your father’s servant that I’m only sitting here to give you this medicine?” So, I stopped going. […] He should’ve spoken kindly. […] [If he had spoken kindly] I would not have left the treatment." - IDI-20 (Forty-two-year-old male LTFU)

Personality and Attitude

Interviews with some patient participants revealed that despite having no external motivators, and in some cases having what could be considered barriers to treatment adherence for others, these participants were still able to complete their treatment. They displayed an inherent discipline which facilitated adhering to each day’s dose and an overall motivation which helped them complete the treatment. Some patient-participants illustrated how they ensured that they never missed a dose - e.g. they used external cues such as mealtimes to time each dose. Many key informants also attributed the patients' ability to complete the treatment course to their degree of determination. In case of LTFUs, the lack of internal determination and discipline led them to succumb to the other causes of LTFU, such as alcohol use.

Further, the patient's attitude towards the health system impacted treatment completion. If the patient did not have faith in the health system and/or in the medicines, they were unlikely to achieve treatment completion. Some community members and healthcare providers stated that there is a tendency to not have faith in free medicines, since something free is sometimes dismissed as being worthless. However, this belief did not seem to be shared by any of the patient-participants.

Further, a fatalistic attitude towards the inevitability of death, usually displayed by elderly patients, especially those with a history of regular alcohol use, also made these patients susceptible to treatment LTFU. 

“I had full faith. It was just about taking medicines. Tell me to take them for six years instead of six months, and I will easily comply. It’s no trouble for me.” - IDI-29 (Sixty-four-year-old male with successful outcome)

Financial Issues

The next major domain which emerged in our study was the effect of the financial burden of TB as a disease. Even though TB medicines and investigations were free, patients and their families had to bear both direct and indirect costs - transport costs, cost of medicines to relieve side effects, cost of dietary supplements, cost of certain investigations, and the loss of wages. About half the key informants felt that the patient’s income was a possible predictor of their treatment outcome. It influenced the diet of the patient as well as their ability to buy medicines for symptomatic relief. This further influenced their ability to tolerate side effects, as well their general well-being. The patient’s financial status also influenced his/her ability to bear the loss of wages she/he incurred during ill health.

Some LTFU patients revealed that financial burden was why they discontinued treatment - most of these LTFU patients were availing treatment from private health sector. Financial constraints had caused them to stop treatment from the private health sector and turn to the public health sector for treatment. Such an interruption may have eased the financial strain but introduced a significant gap in treatment leading to first-time LTFU. By contrast, treatment completers were better equipped to manage the financial burden of their disease, either by virtue of their own household earnings, or with the help of small loans from relatives.

“I was stuck at home, and I was the sole breadwinner. I had little money, would buy few things to eat and drink. Once I ran out of money, I managed by borrowing from a neighbour. If the breadwinner is at home, then who will earn? […] There was no money. I didn’t even get proper diet to go along with the medicines. I could handle the medicines till the time I got proper diet. When the body has strength, it can handle the medicines. […] What will a poor man do, what will he eat? Will he feed his children, or will he feed himself? - IDI-11 (Fifty-one-year-old male LTFU)

Stigma

Most key informants as well as patients-participants felt that social stigma was not a significant determinant of treatment outcome, except in a few cases. In such cases, TB-associated stigma helped, rather than hindered, treatment completion. Families of unmarried girls wanted their daughters to be treated, and thus cured, Families of unmarried girls requested DOTS providers to place the medicines with them. Thus, neither the patient needed to visit the DOTS centre nor the DOTS provider needed to visit the patients’ house. Such an arrangement was envisaged to maintain discreetness in the treatment of tuberculosis and avoid social stigma. Few patients would have preferred a DOTS centre that was away from their community so as to avoid meeting anyone who knew them.

“Several times, family members of a patient have said to us that no one should find out that their child is taking medicines. No one should find out. You keep giving it discreetly, and we will keep having it.” - FGD-4 (DOTS providers)

Health Belief Model

**Figure 1:**
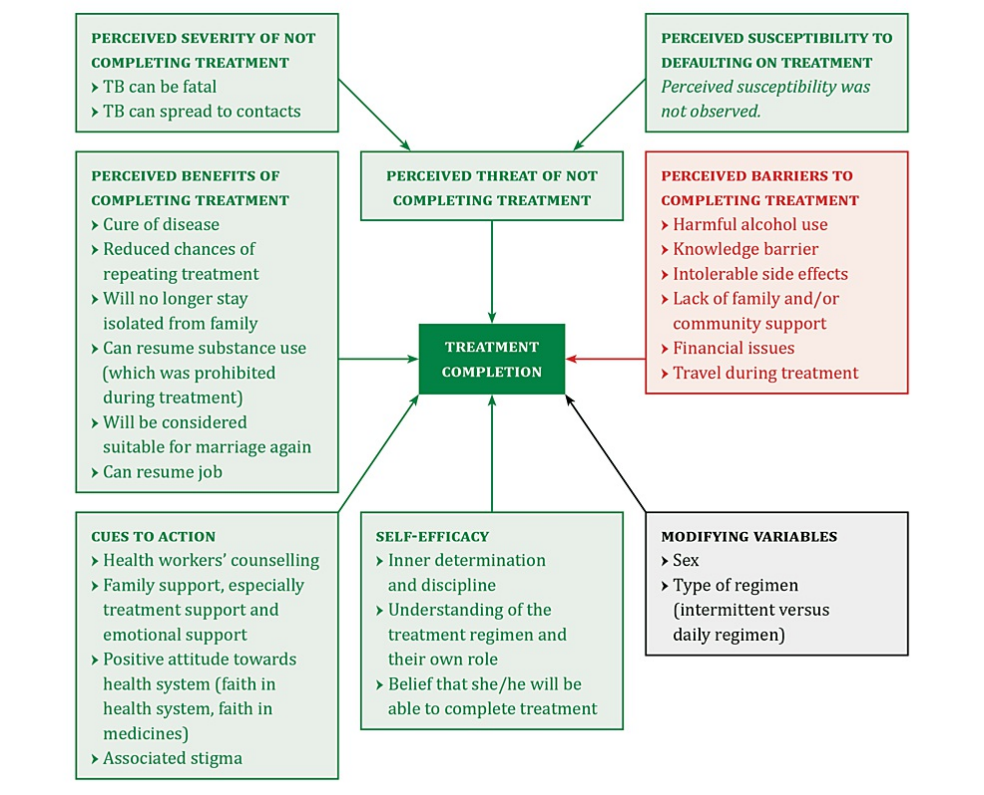
Health Belief Model for Tuberculosis Treatment Completion

Based on the themes identified by analysis of KIIs and FBDs, we constructed an HBM to provide a clearer understanding of the dynamics of tuberculosis treatment LTFU (Figure 1).

## Discussion

We conducted this community-based qualitative study to ascertain the determinants of treatment LTFU in TB patients in the rural and semi-urban areas of north India. The results were based on the perceptions of a variety of stakeholders, including not only patients and healthcare providers, but also members of the community such as Panchayati Raj Institution (PRI) members and members of the general community. During the interview process, various aspects of TB treatment were discussed at length. During data analysis, a grounded theory approach was used to inductively arrive at the broad domains to which the determinants belonged. 

The first key domain identified was external motivators* *i.e. perceived adverse consequences of discontinuing treatment and perceived benefits of treatment completion. Previous Indian studies had not identified this theme as a determinant of LTFU. However, Chida et al. [16], based on their study in Karachi, Pakistan did note that one patient was LTFU because he had no family, and therefore, he felt unmotivated to continue treatment. The meta-ethnography by Munro et al. [19] also reported that patients with a desire for a cure, and/or a fear of the negative consequences of irregular treatment, were more likely to adhere to treatment. Hence, understanding what motivates a patient to complete treatment, and then using that knowledge tactfully throughout treatment may help prevent LTFU.

Knowledge about treatment duration and different treatment outcomes was also an important determinant of treatment outcome. Our findings were similar to those reported by Jaiswal et al. [11], Velavan et al. [15], Shringarpure et al. [14], as well as studies conducted outside India [16,17]. Due to the ubiquitous nature of this domain, it was successfully identified as a determinant of treatment outcome by Munro et al. [19]. A noteworthy finding of our study is that a few participants had been LTFU because they had misinterpreted the negative sputum smear results (at the end of the intensive phase) to mean cure. This highlights the need for proper patient counselling and education at the time of initiation of therapy and at various points during the course of treatment, such as at the end of the intensive phase, continuation phase, and during follow-up.

We found that regular alcohol use was one of the most important determinants of LTFU. Addiction to alcohol as a determinant of LTFU has also been reported by various qualitative studies from India viz. Jaiswal et al. [11], Shringarpure et al. [14], Velavan et al. [15]. However, qualitative studies conducted outside India could not definitively identify regular alcohol use as an important determinant of LTFU. Munro et al. also did not capture it in their meta-ethnography [19]. However, quantitative studies showing a significant association between alcohol use and LTFU were aplenty [21-23]. TB patients with a history of regular alcohol use should be offered appropriate, timely, and repeated counselling as well as de-addiction services.

Further, patients found it difficult to complete the treatment in the absence of family support. This was supported by literature from India and abroad [12,14,15,18,19].* *Munro et al. [19] did report that family support - including financial assistance, collecting medication, and emotional support - strongly influenced adherence, and deemed it important enough to be one of their eight main themes.

About half of the LTFUs in our study had left the treatment because of the ATT-related side effects* a*nd the lack of any provisions (such as medicines or dietary supplements) to alleviate them. Our findings agreed with the results of Jaiswal et al. [11] in Delhi. Outside India, Chida et al. [16] reported that side effects were the most commonly reported primary reason for LTFU in their study setting in Karachi, Pakistan. Munro et al. [19] also identified side effects as one of the eight main themes leading to LTFU.

In our study, all stakeholders agreed that the overall experience with the health system and the behaviour of healthcare providers (HCPs) were crucial determinants of the treatment outcome. The importance of HCPs’ behaviour has been highlighted across the board [11,15,22]. Other studies have also reported the importance of repeated counselling - Velavan et al. [15] reported that patient motivation through individual and family counselling and by sharing peer experiences could be fruitful. Further, HCPs interviewed in our study felt that instead of transferring money, providing free rations would better help patients adhere to treatment. Velavan et al. [15] also concluded that nutritional supplements for deprived patients would improve treatment adherence and response, which may be given as a supplement or bought with the help of the monetary incentives given to them. Shiotani and Hennink [13] also described the unaffordability of the high-protein diet (comprising milk and eggs) recommended by HCPs for patients on medication. These patients perceived the need for food supplements to be provided free of cost by the government treatment programmes for the duration of receiving DOTS.

Similar to our study, others also identified patients' personality and attitude* *to be significant determinants of treatment LTFU. Munro et al. [19] also found that personal agency was an important aspect of adherence behaviour - e.g. those who developed their own reminders adhered readily. Velavan et al [15] noted that ‘defaulters tend to default again,' and that ‘habitual LTFU’ was a potential risk factor for unfavourable outcomes. On attitude towards the health system - Chida et al. [16] reported that most of the patients who were LTFU due to negative health system interactions were dissatisfied with their entire health system and believed that there was no use seeking care elsewhere.

Financial issues as a determinant of treatment LTFU have been previously recognised in literature Velavan et al. [15], who reported that patients who could not afford good nutrition found it difficult to cope with treatment. Jayachandran [12] reported that lack of money was the most common reason for LTFU; she also reported the huge expenditure in the private health sector along with a sense of feeling better after two-three months led to LTFU. Shiotani and Hennink [19] found that lost income caused significant frustration amongst male patients of tribal ethnicity, leading to infrequent treatment visits and treatment LTFU. Half the sample interviewed by Chida et al. [16] reported finances as a primary or secondary reason for LTFU, making it the most common theme referenced in their study. Hasker et al. [18] also reported a loss of income and transport costs as barriers to compliance. Moreover, they reported that reimbursement of transport costs and the provision of supplementary food items were important enabling factors identified by treatment completers interviewed in their study. Patients also expressed guilt over the impact of the disease on their family livelihoods. Males (usually the head of households and/or the sole wage earners) tended to cite financial issues as the reason for LTFU more frequently than females [15].

Overall, there was no indication that stigma was a significant determinant of the treatment LTFU. However, social stigma against TB could affect one’s social standing within the community, and especially affect one’s marriage prospects. We found that families of young unmarried girls wanted their daughters to complete treatment at home so that they would be considered suitable for marriage. This was in consonance with the findings of Shringarpure et al. [14] but in contrast to the findings of Shiotani and Hennink [13], who reported that though a few patients would endure the risk of stigma to complete treatment, other patients’ attempts to conceal their disease status reduced adherence - they “would hide medicines and would cease taking them as soon as they felt better, rather than completing the course, leading to treatment default”.

An HBM [20] was constructed to understand the perceived threats, benefits and barriers to treatment completion. It suggests that if the perceived severity of treatment discontinuation is high, then the patient is more likely to complete the treatment. The primary external motivator is “fear”, which in turn contributes to the perceived threat component of HBM. The perceived susceptibility is not a strong factor in our HBM since none of the patients considered themselves inherently susceptible to LTFU. A perceived barrier to treatment completion is the misinterpretation of symptomatic relief as 'cure'. Specific cues to action facilitate the process of treatment completion. These include support and repeated counselling from both families as well as the health system.

Strengths

We ensured the participation of all relevant stakeholders, i.e. patients, healthcare providers, policymakers, middle- and lower-level program managers, and implementers, as well as representatives of the general community, from both urban and rural areas. All potential participants who were approached agreed to participate. The refusal rate was nil. All interviews were conducted by a single investigator (AK). Thus, inter-observer variation was avoided.

The entire qualitative data analysis was done manually and in Hindi (the language in which interviews were conducted). Hence, no nuances were lost in translation. The coding, identification of themes, and development of HBM were done repeatedly to ensure that none of the biases of the investigator who collected the data crept into the results. The final results were based on the triangulation of data from various stakeholders, and from both IDIs as well as FGDs, which led to an increased level of dependability and confirmability of the findings.

The final results were based on the triangulation of data from various stakeholders, and from both IDIs as well as FGDs, which led to an increased level of dependability and confirmability of the findings. The entire study has been transparently reported as per the COREQ guidelines (Appendix 1).

Limitations

Like all qualitative research, this study could also have limitations of purposive and convenient sampling. The findings may not be transferable to other locations. 

## Conclusions

There are several important determinants of treatment loss-to-follow-up in TB patients. One major determinant was the effect of harmful use of alcohol on treatment completion. Other determinants of note are external motivators, the financial burden of treatment, side effects of ATT drugs, and the level of support from the family. The results of this study may help in developing a framework to identify patients at high risk of LTFU, so that pre-emptive steps can be taken. The journey from treatment initiation to treatment completion is often extremely traumatic for the patient. Repeated counselling of not just the patient but also her/his family, with content that is scientifically sound as well as customised to the unique situation of each patient, is the need of the hour. After all, the onus to successfully complete treatment lies not with the patient alone, but with the health system as well.

## Appendices

Appendix 1 

**Table 3:** COREQ (COnsolidated criteria for REporting Qualitative research) Checklist

No. Item	Item No.	Guide questions/ description	Reported in section/ remarks
Domain 1: Research team and reflexivity			
Personal Characteristics			
Interviewer/ facilitator	1	Which author/s conducted the interview or focus group?	Materials and methods
Credentials	2	What were the researcher’s credentials? E.g. PhD, MD	Materials and methods
Occupation	3	What was their occupation at the time of the study?	Materials and methods
Gender	4	Was the researcher male or female?	Materials and methods
Experience and training	5	What experience or training did the researcher have?	Materials and methods
Relationship with participants			
Relationship established	6	Was a relationship established prior to study commencement?	Materials and methods – Data collection
Participant knowledge of the interviewer	7	What did the participants know about the researcher? e.g. personal goals, reasons for doing the research	Materials and methods – Data collection
Interviewer characteristics	8	What characteristics were reported about the interviewer/ facilitator? e.g. Bias, assumptions, reasons and interests in the research topic	Materials and methods – Data collection
Domain 2: Study design			
Theoretical framework			
Methodological orientation and Theory	9	What methodological orientation was stated to underpin the study? e.g. grounded theory, discourse analysis, ethnography, phenomenology, content analysis	Materials and methods – Data analysis
Participant selection			
Sampling	10	How were participants selected? e.g., purposive, convenience, consecutive, snowball	Materials and methods – Study setting and study participants
Method of approach	11	How were participants approached? e.g. face-to-face, telephone, mail, email	Materials and methods – Data collection
Sample size	12	How many participants were in the study?	Results
Non-participation	13	How many people refused to participate or dropped out? Reasons?	Results
Setting			
Setting of data collection	14	Where was the data collected? e.g. home, clinic, workplace	Materials and methods – Data collection
Presence of non-participants	15	Was anyone else present besides the participants and researchers?	Materials and methods – Data collection
Description of sample	16	What are the important characteristics of the sample? e.g. demographic data, date	Results
Data collection			
Interview guide	17	Were questions, prompts, guides provided by the authors? Was it pilot tested?	Materials and methods – Development of tools
Repeat interviews	18	Were repeat interviews carried out? If yes, how many?	Results
Audio/ visual recording	19	Did the research use audio or visual recording to collect the data?	Materials and methods – Data collection
Field notes	20	Were field notes made during and/or after the interview or focus group?	Materials and methods – Data collection
Duration	21	What was the duration of the interviews or focus group?	Results
Data saturation	22	Was data saturation discussed?	Results
Transcripts returned	23	Were transcripts returned to participants for comment and/ or correction?	Yes, transcripts were discussed with all participants who could be recontacted.
Domain 3: Analysis and findings			
Data analysis			
Number of data coders	24	How many data coders coded the data?	Materials and methods – Data analysis
Description of the coding tree	25	Did authors provide a description of the coding tree?	Materials and methods – Data analysis
Derivation of themes	26	Were themes identified in advance or derived from the data?	Materials and methods – analysis; Results
Software	27	What software, if applicable, was used to manage the data?	Material and methods – analysis (Manual analysis)
Participant checking	28	Did participants provide feedback on the findings?	Yes, partially. All key informants provided feedback on the findings. Patient-participants could not be revisited due to logistical limitations (Repeating travel of approximately 350 km with 2-3 visits per patient was deemed infeasible).
Reporting			
Quotations presented	29	Were participant quotations presented to illustrate the themes/ findings? Was each quotation identified? e.g., participant number	Results – Determinants of treatment loss-to-follow-up
Data and findings consistent	30	Was there consistency between the data presented and the findings?	Results
Clarity of major themes	31	Were major themes clearly presented in the findings?	Results
Clarity of minor themes	32	Is there a description of diverse cases or discussion of minor themes?	Results
